# Combinatory Effects of Cerium Dioxide Nanoparticles and Acetaminophen on the Liver—A Case Study of Low-Dose Interactions in Human HuH-7 Cells

**DOI:** 10.3390/ijms22136866

**Published:** 2021-06-25

**Authors:** Benjamin C. Krause, Fabian L. Kriegel, Victoria Tartz, Harald Jungnickel, Philipp Reichardt, Ajay Vikram Singh, Peter Laux, Mohamed Shemis, Andreas Luch

**Affiliations:** 1 Department of Chemical & Product Safety, German Federal Institute for Risk Assessment, Max-Dohrn-Straße 8-10, 10589 Berlin, Germany; Fabian.Kriegel@nuvisan.com (F.L.K.); Vic.Tartz@outlook.de (V.T.); Harald.Jungnickel@bfr.bund.de (H.J.); Philipp.Reichardt@bfr.bund.de (P.R.); Ajay-Vikram.Singh@bfr.bund.de (A.V.S.); Peter.Laux@bfr.bund.de (P.L.); Andreas.Luch@bfr.bund.de (A.L.); 2 NUVISAN ICB GmbH, Preclinical Compound Profiling, Muellerstrasse 178, 13353 Berlin, Germany; 3 Department of Biochemistry & Molecular Biology, Theodor Bilharz Research Institute, Warak El-Hadar, Kornish El-Nile, P.O. Box 30 Imbaba, Giza 12411, Egypt; M.Shemies@tbri.gov.eg

**Keywords:** ToF-SIMS, HuH-7 cells, acetaminophen, nanoparticles, cerium dioxide, metabolomics, liver

## Abstract

The interactions between pharmaceuticals and nanomaterials and its potentially resulting toxicological effects in living systems are only insufficiently investigated. In this study, two model compounds, acetaminophen, a pharmaceutical, and cerium dioxide, a manufactured nanomaterial, were investigated in combination and individually. Upon inhalation, cerium dioxide nanomaterials were shown to systemically translocate into other organs, such as the liver. Therefore we picked the human liver cell line HuH-7 cells as an in vitro system to investigate liver toxicity. Possible synergistic or antagonistic metabolic changes after co-exposure scenarios were investigated. Toxicological data of the water soluble tetrazolium (WST-1) assay for cell proliferation and genotoxicity assessment using the Comet assay were combined with an untargeted as well as a targeted lipidomics approach. We found an attenuated cytotoxicity and an altered metabolic profile in co-exposure experiments with cerium dioxide, indicating an interaction of both compounds at these endpoints. Single exposure against cerium dioxide showed a genotoxic effect in the Comet assay. Conversely, acetaminophen exhibited no genotoxic effect. Comet assay data do not indicate an enhancement of genotoxicity after co-exposure. The results obtained in this study highlight the advantage of investigating co-exposure scenarios, especially for bioactive substances.

## 1. Introduction

Drug-induced liver injury (DILI) is a common cause for acute liver failure, affecting not only patients, but also drug developers, drug regulating authorities and health care providers [1,2]. The best-known model compound for DILI is acetaminophen (APAP), which is often subscribed as an oral antipyretic and analgesic [3]. APAP overdose causes severe liver toxicity and may even result in death [4]. The toxicity of APAP is caused via the generation of the reactive *N*-acetyl-*p*-benzoquinone imine (NAPQI) during phase I of hepatic biotransformation, followed by cell necrosis and/or apoptosis of the hepatocytes exposed [1]. The development of DILI may also be influenced by chemical mixtures or substances occurring as nanoparticles (NPs). NPs are known to cause toxicity via the generation of reactive oxygen species (ROS) [5] and may, therefore, affect the pathogenesis of DILI. A frequently applied NP in several consumer product areas is cerium dioxide (CeO_2_) [6]. CeO_2_ based nanocarriers were already successfully employed as a inflammatory regulator by utilizing their scavenging activity of ROS in DILI [7]. In daily life, CeO_2_ is commercially used in lacquers and wood-protection coatings [8]. Furthermore, nano-scaled CeO_2_ is applied in exhaust gas catalysts of cars and vans taking advantage of its unique oxygen-storing capability. The substance is also used as a diesel fuel additive to reduce fuel consumption and soot particle emission of diesel engines [9,10].

The main exposure route for nano-sized CeO_2_ is via inhalation. Inhalation of NPs is associated with dose-dependent adverse effects on the respiratory tract [11]. In a 5-day inhalation study rats were exposed to 0.5, 2.5 or 10 mg/m^3^ CeO_2_. Here, a dose-dependent inflammation of pulmonary tissue could be observed, with effects already emerging at the lowest dose applied [12]. A 2-year chronic inhalation in vivo study applying CeO_2_ (NM-212) NPs in Wistar rats showed the accumulation of CeO_2_ NPs in liver tissue [13]. However CeO_2_ is a known antioxidant which showed favourable effects when combined with compounds that induce ROS. In vivo experiments applying CeO_2_ in combination with APAP highlight the protective effect. Nevertheless the nanoparticulate nature of CeO_2_ may also contribute to a non-physiological environment [14]. In principle, CeO_2_ may interfere directly with a drug or cause additional damage through the induction of ROS, thus possibly accelerating the adversity of the drug metabolites. This notion points to the necessity of looking into co-exposure scenarios of NPs with pharmaceuticals or their active metabolites.

In this study we exposed the human liver cell line HuH-7 to subtoxic concentrations of APAP and/or CeO_2_ NPs. After NP characterization using dynamic light scattering (DLS), nanoparticle tracking analysis (NTA) and single particle inductively coupled plasma mass spectrometry (SP-ICP-MS), toxicological endpoint assays such as the water soluble tetrazolium (WST-1) and Comet assay were applied and supplemented with a targeted metabolomics approach. In addition, time-of-flight secondary ion mass spectrometry (ToF-SIMS) was applied as an untargeted metabolomics approach to assess cell membrane changes upon treatment of cells.

## 2. Results and Discussion

### 2.1. Particle Characterization

The NPs used in this study (NM212, JRC) were comprehensively investigated by the manufacturer as well as in the frame of several research projects [13,15]. A detailed transmission electron microscopy (TEM) characterization containing further information about size and morphology of the applied reference nanomaterial CeO_2_ (NM212) can be found in the corresponding Joint Research Centre (JRC) report (for details, see [15]). To look into possibly altered physicochemical characteristics due to interactions of CeO_2_ NPs and APAP, different sizing techniques were applied. DLS data, however, indicated no altered hydrodynamic diameters of particles when combining CeO_2_ with APAP (see Table 1). In fact, our results of ~210 nm are in good accordance with the manufacturers results of ~213 nm [15]. On the other hand, NTA analysis revealed a shift to higher particle sizes and a decreasing particle number concentration with increasing APAP concentrations (see Table 1).

**Figure 1:**
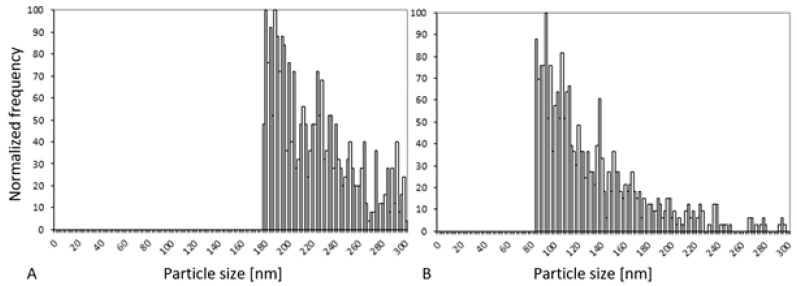
Particle size distribution of 400 ng mL^−1^ CeO_2_ NPs with (**A**) 0.5 mmol L^−1^ and (**B**) 50 mmol L^−^^1^ APAP as determined by single particle inductively coupled plasma mass spectrometry (SP-ICP-MS).

Both DLS and NTA determine the hydrodynamic diameter in situ but cannot differentiate between particles, proteins and other scattering substances. Therefore, SP-ICP-MS (see Figure 1) was used to analyze changes in the core particle size of CeO_2_ NPs after mixing with APAP. The detection limit for SP-ICP-MS for such complex samples limits the significance of the obtained data. Nevertheless, an increase in APAP concentration shifts the main peak of the particle size distribution to a lower size range of about 80–100 nm. Those findings highlight a potential interaction of CeO_2_ NPs and APAP, which led to smaller particles.

### 2.2. WST-1 Assay

To be able to elucidate non-apoptotic alterations of the metabolome only, a WST-1 assay was conducted to determine subtoxic exposure conditions. A sufficient threshold was concluded to be the WST-1 readout above 50%.

A known issue in colorimetric assays are interactions of NPs with other test chemicals. To account for these interactions, a control suspension of particles was subjected to the WST-1 assay in the absence of cells. The obtained result of only 0.1% readout compared to control was considered negligible.

**Figure 2:**
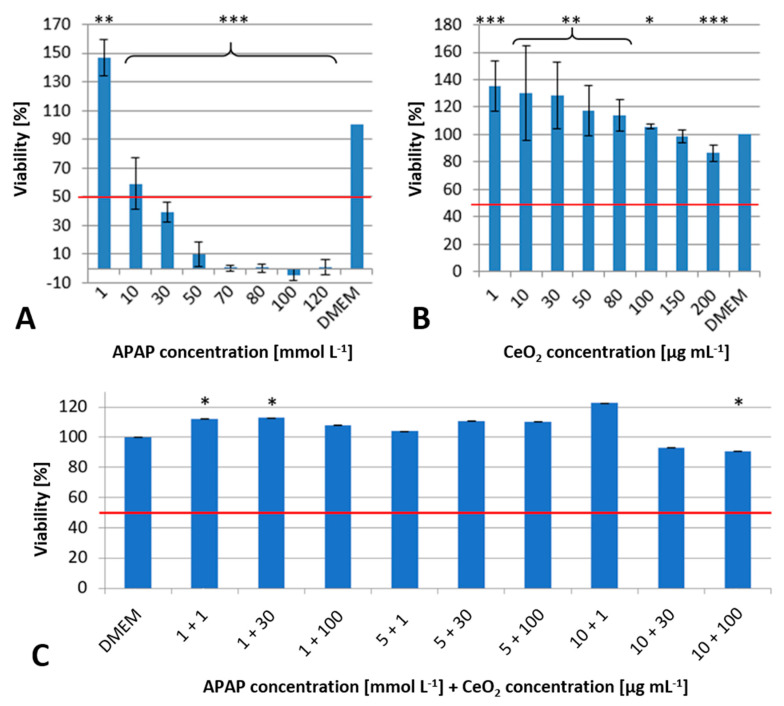
Results of the WST-1 assay after 24 h exposure of HuH-7 cells to APAP (**A**), CeO_2_ NPs (**B**) and both in combination (**C**) at different concentrations ± standard deviation normalized to negative control (Dulbecco’s modified Eagle medium, DMEM) and positive control (TritonX). Red line indicates the EC50 (effective concentration where viability is reduced to 50%). All tests with at least three biological and six technical replicates. *p* ≤ 0.05 = *, 0.01 = **, 0.001 = ***.

Whilst in Figure 2A, the lowest APAP concentration of 1 mmol L^−1^ causes an increase in cell viability in both cell types, it decreases by at least 50% at a concentration of 10 mmol L^−1^. At a concentration of 30 mmol L^−1^, the LD50 is exceeded, and the viability of both cells decreases to values below 50%. If the APAP concentration is further increased, the viability of the cells is not detectable.

Upon exposure to CeO_2_ NPs, a slight dose-dependent decrease of the viability is observed (Figure 2B). However, the initial viability exceeds the values of the negative control (Dulbecco’s modified Eagle medium, DMEM). Up to a dose of 200 µg mL^−1^ the LD50 is not reached. It is known that the redox potential of CeO_2_ has a protective effect in vitro due to the superoxide dismutase-like functionality [16].

The control-related viability after combined exposure shown in Figure 2C demonstrated no consistent trend in the effects of the NP and APAP for all investigated concentrations. Only the application of the highest concentrations (APAP: 10 mmol L^−1^, CeO_2_ NP: 30 and 100 µg mL^−1^) showed a reduction of viability by about 10%.

However, the co-exposure of both substances suppressed the toxicity of APAP even at concentrations which exceeded the LD50 for single exposure of APAP. A possible explanation for the reduced toxicity in co-exposure may be an interaction of CeO_2_ with APAP leading to a partially inaccessibility of APAP for CYP2E1. The process may prevent the formation of the reactive metabolite *N*-acetyl-*p*-benzoquinone imine. This interaction could on the other hand cause the formation of larger agglomerates, which hinder the uptake into the cells.

**Figure 3:**
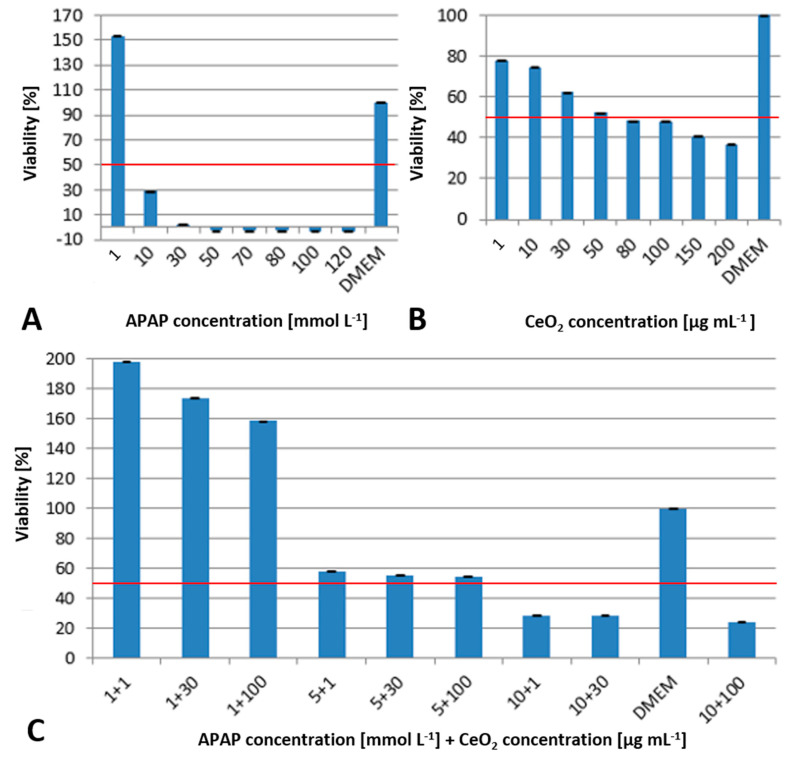
Results of the WST-1 assay after 48 h exposure of HuH-7 cells to APAP (**A**), CeO_2_ NPs (**B**) and both in combination (**C**) at different concentrations ± standard deviation normalized to negative control (DMEM) and positive control (TritonX). Red line indicates the EC50. All tests with one biological and six technical replicates.

To assess a possible time-dependent uptake and toxicity mechanism, WST-1 assays were also conducted after 48 h of incubation. Co-exposure of HuH-7 cells to APAP and CeO_2_ NPs for 48 h (Figure 3) showed an increased toxicity of both substances. While an APAP concentration of 1 mmol L^−1^ continues to increase viability, the EC50 is already exceeded at 10 mmol L^−1^ (see Figure 3A). When exposed to CeO_2_ NPs, an EC50 value of about 50 µg mL^−1^ and a rising toxicity with increasing concentration was observed (Figure 3B).

A strong viability enhancing effect of 150–200% could be observed at the lowest APAP concentration used in combination with CeO_2_ (see Figure 3C). The viability decreases strongly with increasing APAP concentrations but remained largely unaffected by changes of NP concentrations (see Figure 3C).

The results obtained in the WST-1 assay after 48 h indicate that the suppression of the APAP toxicity in co-exposure with CeO_2_ NP is only temporary. That might be due to the fact that the antioxidant capacity of CeO_2_ is limited and cannot keep pace with the ROS generation of APAP. Since the effective uptake of NPs is size-dependent [17] the co-exposure scenario foster agglomeration (see Section 3.3 and Section 3.5 for uptake and ToF-SIMS investigations). This process delays the uptake of larger agglomerates. After 48 h the agglomerates taken up are sufficient to induce the observed cytotoxic effects (see Figure 2 and Figure 3). Another hypothesis for the observed differences in the WST-1 assay could be the progression of cellular degradation processes causing the dissociation of the formed CeO_2_-APAP complexes, as shown by ToF-SIMS data (see Section 3.5). The resulting non-associated APAP could subsequently be toxified and induce the observed effects.

### 2.3. Cellular Uptake of CeO_2_ Nanoparticles (NPs)

Investigating the cellular uptake of CeO_2_ NPs might give insights in the alterations of cell viability (see Section 3.2). ICP-MS was used to determine the concentration of CeO_2_ NP absorbed in HuH-7 cells after 24 h of exposure. To validate this method for the measurement of an intracellular CeO_2_ NP content, the limit of detection (LOD) and limit of quantification (LOQ) was determined prior to actual cell analysis.

The values of LOD and LOQ determined via the calibration curve method according to DIN 32,645 were 0.006 ng mL^−1^ and 0.02 ng mL^−1^ in DMEM for low and high-dose APAP (0.5 and 50 mmol L^−1^, see Electronic Supplementary Information (ESI) 2), respectively. The values obtained represent a basis for the measurements of intracellular particle contents.

**Figure 4:**
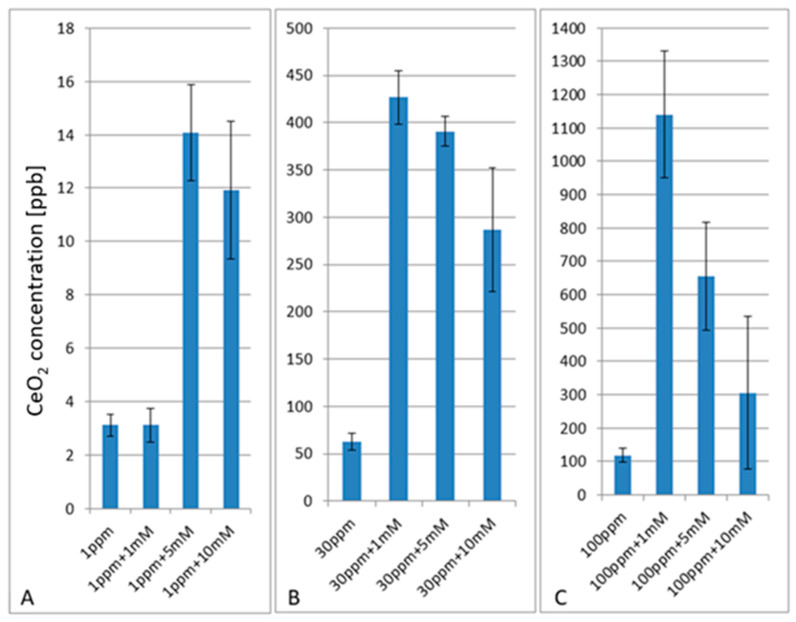
Intracellular CeO_2_ concentrations [ppb = ng mL^−1^] (**A**) measured by ICP-MS of HuH-7 cells upon exposure to CeO_2_ NPs [ppm = µg mL^−1^] (**B**) without or with APAP [mM = mmol L^−1^] (**C**) at different concentrations.

Intracellular concentrations of CeO_2_ NPs in HuH-7 cells were measured by ICP-MS upon exposure to particles alone or along with APAP at different concentrations (Figure 4). A concentration-dependent uptake of particles into the cells could be demonstrated. Simultaneous application of APAP resulted in up to 11 times the intracellular particle concentration when compared to the situation when APAP was absent (see Figure 4). Our ICP-MS data are in contrast to the aforementioned hypothesis that co-exposure of cells against CeO_2_ NPs and APAP would negatively interfere with any particle uptake due to agglomeration.

In fact, the increased uptake of particles upon co-exposure suggests a synergistic uptake mechanism. Previously we could demonstrate that exposure of cells to NPs can induce changes of the cellular membrane composition [18].

Since no increased toxicity could be seen in the WST-1 assay after 24 h of co-exposure, and an increased uptake of CeO_2_ NPs was detected, we further investigated the genotoxic potential of the single and co-exposure scenario. The assessment of DNA damage was carried out based on the alkaline Comet assay.

### 2.4. Genotoxicity Assessment of CeO_2_ NPs in the Absence or Presence of Acetaminophen (APAP)

**Figure 5:**
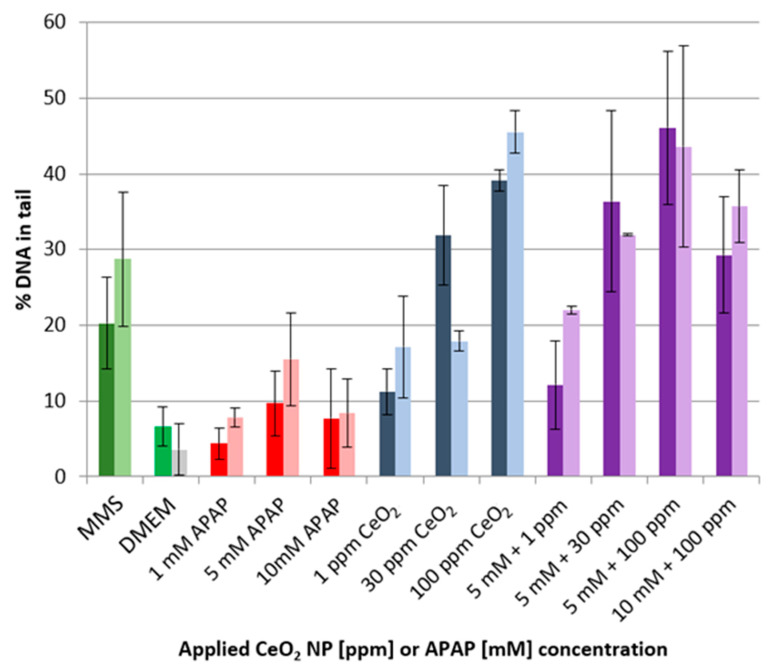
Alkaline Comet assay results with methyl methanesulfonate (MMS) and DMEM as positive and negative control, respectively. DNA damage was quantified as percentage of DNA in the comet tail after 24 (dark colors) and 48 h (light colors), *n* = 2.

The genotoxicity of CeO_2_ NPs in HuH-7 cells was determined either in the presence or the absence of APAP (see Figure 5). The assessment is based on the quantification of DNA in the comet tail. The incubation of the cells with APAP at concentrations of 1, 5 and 10 mmol L^−1^ showed only marginally increased DNA content in the comet tails when compared to the negative control (DMEM). The deviations were non-significant. By contrast, exposure to CeO_2_ NPs caused DNA levels of up to 44% in the comet tails in a particle concentration-dependent manner. The levels observed when both substances were combined corresponded well to the extent seen with NPs alone (no influence of APAP on CeO_2_-mediated genotoxicity). Higher APAP concentrations were shown to reduce the uptake of CeO_2_ NP into the cell (see Figure 4), as proven by ICP-MS.

### 2.5. Time-of-Flight Secondary Ion Mass Spectrometry (ToF-SIMS)

To investigate the uptake of CeO_2_ NPs with or without co-exposure to APAP, along with possible metabolic changes within the cells exposed, we treated HuH-7 cells with CeO_2_ NPs (100 µg mL^−1^) and APAP (5 mmol L^−1^) only, or in combination (5 mmol L^−1^ APAP + 100 µg mL^−1^ CeO_2_ NPs) for 24 h. Subsequently we analyzed the cells by imaging mass spectrometry (ToF-SIMS) to assess the intracellular NP distribution patterns and possible cell membrane changes.

The mass spectra of the ToF-SIMS depth profiles of HuH-7 cells exposed to APAP only (see ESI 3a-c) revealed particles consisting of metal hydroxide complexes (APAP-FeO(OH), APAP-Zn(OH)_2_ and APAP-MnO(OH)_2_). The heavy metals (iron, zinc and manganese) and their metal oxides were also present in the culture medium. These data point to metal oxide APAP complexes as the predominant form of paracetamol storage within HuH-7 cells. Cells exposed to APAP and CeO_2_ NPs contained CeO_2_ NP aggregates (see ESI 3d) as well as APAP-CeO(OH) complexes (see ESI 3e). This kind of chemical transformation suggests heteroagglomerate formation also in the case of non-standard metal oxides industrially produced as manufactured NPs. Presumably, our results suggest, that APAP-metal oxide complexes may play a role in drug induced liver toxicology. The presence of APAP-metal oxides may facilitate APAP storage and its resistance against metabolic degradation in liver cells at least for some time.

**Figure 6:**
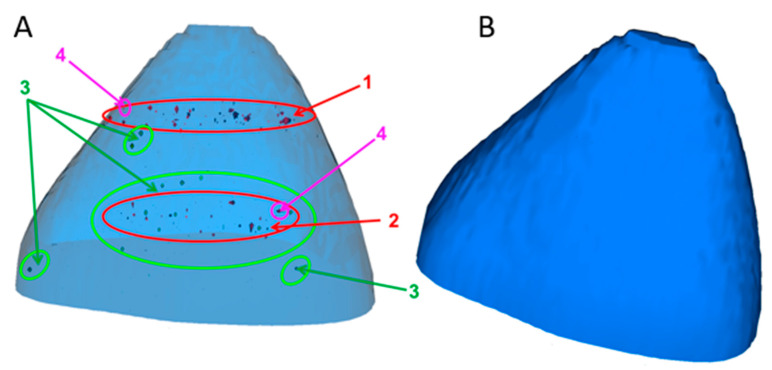
Time-of-flight secondary ion mass spectrometry (ToF-SIMS) ion reconstruction of the 3D depth profile (20 µm × 15 µm × 3.5 µm) of a single HuH-7 cell, which was co-exposed to CeO_2_ NPs (100 µg mL^−1^) and 5 mmol L-1 APAP for 24 h. CeO_2_ NP aggregates are shown in red color (red circle), APAP-CeO(OH) particles are shown in green color (green circle). 1 and 2 (red) indicates the intracellular location of CeO_2_ NPs agglomerates, 3 (green) indicates the intracellular localization of APAP-CeO(OH) particles, whilst 4 (pink) indicates the localization of mixed agglomerates of CeO_2_ NPs and APAP-CeO(OH) particles. The cell membrane is being visualized in translucent (**A**) or solid (**B**) blue and reconstructed from the C_3_H_8_N+ signal that originates from phosphatidylcholine.

The ToF-SIMS 3D reconstruction of a single HuH-7 liver cell co-exposed to CeO_2_ NPs (100 µg mL^−^^1^) and 5 mmol L^−1^ APAP unambiguously indicates the intracellular presence of both CeO_2_ NP agglomerates and APAP-CeO(OH) particles (see Figure 6). Visible are two ring-shaped distinct regions within the cell, where particles accumulate. One region in ca. 900 nm depth within the cell, containing mostly irregular CeO_2_ nanoparticle agglomerates and a second region, which is in about 2.7 µm depth within the cell, which contains both the irregular CeO_2_ NP agglomerates and the irregular but also rod-like APAP-CeO(OH) particles (see Figure 6).

**Figure 7:**
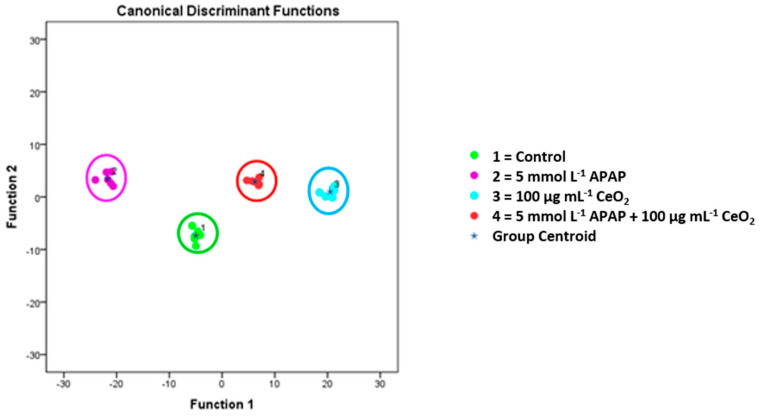
Metabolic changes of cell membrane lipid patterns of HuH-7 cells after treatment with APAP (5 mmol L^−1^), CeO_2_ NPs (100 µg/mL) and APAP (5 mmol L^−1^) plus CeO_2_ NPs (100 µg/mL), as identified by means of ToF-SIMS in combination with multivariate data analysis. The diagram shows the values of the discriminant scores obtained from Fisher’s discriminant analysis of 24 HuH-7 samples. The model was evaluated using the “leave-one-out” formalism (100% correct grouping of ungrouped cases).

Additionally, metabolic alterations upon treatment were assessed in HuH-7 cells using ToF-SIMS. The results show significant differences between HuH-7 cell membrane lipid patterns for cells exposed to 5 mmol L^−1^ APAP, cells exposed to 100 µg mL^−1^ CeO_2_ and cells co-exposed to 5 mmol L^−1^ APAP and 100 µg mL^−1^ CeO_2_ (see Figure 7).

**Figure 8:**
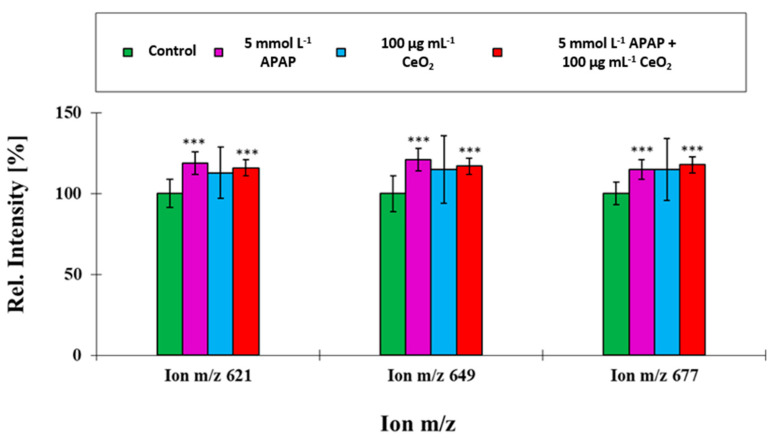
Comparison of ion yields for characteristic cell membrane lipids obtained from the co-exposure experiment with APAP (5 mmol L^−1^) and CeO_2_ NPs (100 µg mL^−1^). These ions were used to separate the four treatment groups. For the relative intensity, the mean of the control group for unexposed HuH-7 cells was taken as 100% in all cases. ***: *p* ≤ 0.05.

**Figure 9:**
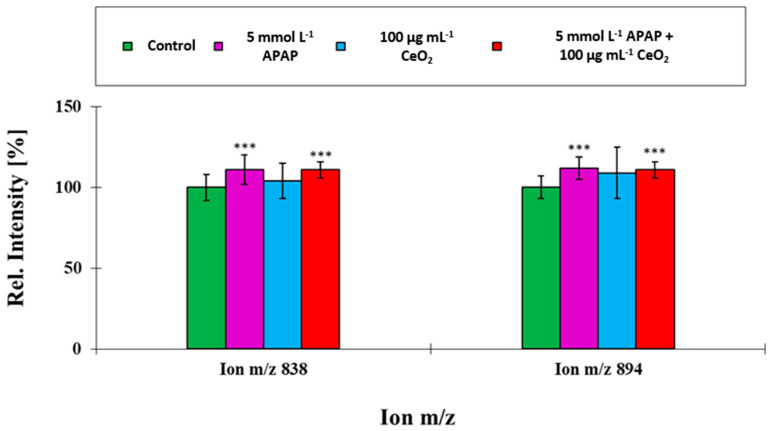
Comparisons of ion yields for characteristic cell membrane lipids obtained from the co-exposure experiment with APAP (5 mmol L^−1^) and CeO_2_ NPs (100 µg mL^−1^). These ions were used to separate the four treatment groups. For the relative intensity, the mean of the control group for unexposed HuH-7 cells was taken as 100% in all cases. ***: *p* ≤ 0.05.

For compounds, which loaded high on factor 1 and hence are mainly responsible for group separation, the data revealed non-additive behavior in co-exposure experiments. A striking feature is the presence of palmitic acid in the two phospholipid groups which directed the separation, that are, sphingomyelins (SM) and phosphatidylinositols (PI). The results (see Figure 8 and Figure 9) show that a series of SM (d12:0/C16:0)—ion m/e 621, SM (d14:0/C16:0)—ion m/e 649 and SM (d16:0/C16:0)—ion m/e 677 and the PIs (C16:0/C18:1) and (C16:0/C20:1), all containing palmitic acid (C16:0) direct the separation. All five compounds were elevated for HuH-7 cells, which were exposed to 5 mmol L^−1^ APAP in the co-exposure experiments. Previous studies of altered lipid patterns in DILI patients showed also elevated levels of palmitic acids [19]. Enhanced palmitate levels are associated with reactive oxygen species and endoplasmic stress as well as a subsequent activation of the c-Jun N-terminal kinases (JNK) c pathway followed by a proinflammatory response [20,21,22]. It is therefore concluded that the alterations in the lipid profile are based on the adverse effects caused by APAP. This is furthermore supported by the increased levels of SM which are associated with enhanced levels of oxidative stress [23] and could be only identified in APAP samples (see Figure 8).

Only cells exposed to APAP alone or in combination with CeO_2_ NPs contained significantly increased PI levels (see Figure 9). The increase of PI might also lead to an enhanced synthesis of diacylglycerols which is suspected to be a key event in the development of fibrosis [24].

Taken together our ToF-SIMS lipidomics data suggest the induction of DILI due to APAP treatment alone or in combination with CeO_2_ NPs.

### 2.6. Targeted Metabolomics

A targeted metabolomics approach was chosen to gain deeper insights into metabolic alterations. The Absolute IDQ p180 kit was utilized to look into a selection of metabolomics data retrieved from whole HuH-7 cells. The data obtained were evaluated by multivariate statistics.

**Figure 10:**
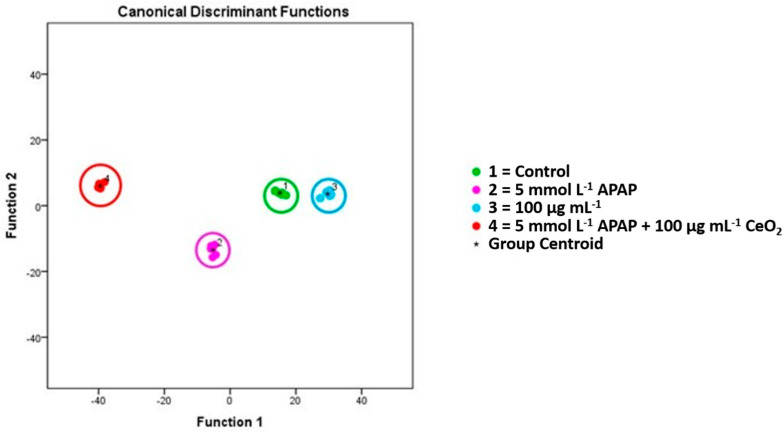
Metabolic changes of HuH-7 cells after treatment with APAP (5 mmol L^−1^), CeO_2_ NPs (100 µg mL^−1^) and APAP (5 mmol L^−1^) plus CeO_2_ NPs (100 µg mL^−1^). The diagram shows the values of the discriminant scores obtained from Fisher’s discriminant analysis of 24 HuH-7 samples for 78 principal compounds, which were selected to discriminate between cells treated with APAP (5 mmol L^−1^), CeO_2_ NPs (100 µg mL^−1^) and APAP (5 mmol L^−1^) plus CeO_2_ NPs (100 µg mL^−1^). All four groups can be well separated from each other. The model was evaluated using the “leave-one-out” formalism (100% correct grouping of ungrouped cases).

Comparing metabolic profiles between cells treated with APAP (5 mmol L^−1^) alone, CeO_2_ NPs alone (100 µg mL^−1^), APAP (5 mmol L^−1^) together with CeO_2_ NPs (100 µg mL^−1^), and untreated cells resulted in 78 cellular metabolites which could be used in a multivariate model to separate between these four treatment groups (see Figure 10).

**Figure 11:**
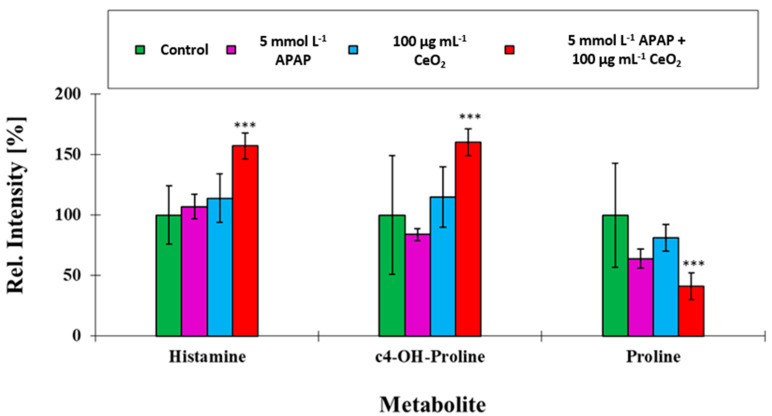
Comparison of ion yields for characteristic biomarker metabolites addressed in the targeted metabolomics experiment of cells co-exposed to APAP (5 mmol L^−1^) and CeO_2_ NPs (100 µg mL^−1^). These metabolites were used to separate the four treatment groups. The mean of the control group of unexposed HuH-7 cells was taken as 100% in all cases. ***: *p* ≤ 0.05.

The results revealed for those compounds, which loaded high on PCA factor 2, non-additive behavior under co-exposure conditions. Two among these cellular metabolites, which had the highest loading on factor 2, namely histamine and c4-OH-proline, were synergistically affected under co-exposure (*p* ≤ 0.05), whereas the levels of proline were antagonistically affected (*p* ≤ 0.05) (see Figure 11).

Histamine levels were significantly elevated in the co-exposure. Histamine is known to be built by decarboxylation of histidine [25], and to contribute to local tissue inflammation due to its pro-inflammatory potential [26]. Co-exposition of APAP and CeO_2_ NPs seems to change the intracellular metabolic pathways for amino acid biosynthesis. While ToF-SIMS analysis gave hints for an increased toxicity of APAP, its combination with CeO_2_ NPs fostered cellular stress and pro-inflammatory processes.

The targeted metabolomics data obtained showed a significant increase of hydroxyproline in cells co-exposed to APAP and CeO_2_ NPs. Hydroxyproline is a known biomarker for induction of fibrosis in liver cells [27]. The results showed that increased levels of hydroxyproline directly correlate with the stage of liver fibrosis [27]. High hydroxyproline levels suggest a metabolic impairment of the hepatocytes and the induction of adverse effects like fibrosis and metabolic reprogramming.

## 3. Materials and Methods

### 3.1. Chemicals

APAP was purchased in analytical standard quality (Sigma Aldrich, St. Louis, MO, USA) and prepared as a 10 M stock solution in DMSO (Sigma Aldrich, St. Louis, MO, USA). CeO_2_ NP (NM212, ~28 nm, JRC Joint Research Centre, Ispra, Italy) were dispersed according to the modified NanoGenoTox protocol and as previously described [28]. In brief, a 2.56 mg mL^−1^ stock dispersion in 0.05% bovine serum albumin (BSA) was prepared and dispersed using an ultrasonic tip sonicator (Bandelin, Berlin, Germany) applying an energy of 1176 kJ mL^−1^ dispersion using an acoustic power of 7.35 W. The stock solution was then diluted in DMEM to achieve final test concentrations. BSA was bought from Sigma Aldrich (Sigma Aldrich, St. Louis, MO, USA). Cell culture medium (DMEM, supplemented with high glucose (4.5 g L^−1^), sodium pyruvate; L-glutamine; 1% penicillin/streptomycin (P/S), and fetal bovine serum) was used for cell culture experiments. All other chemicals used in this study were reagent grade.

### 3.2. Dynamic Light Scattering (DLS)

The distributions of the hydrodynamic diameters of the NPs were determined using a Malvern Nano ZS (Malvern Inc., Malvern, UK). 500 µL of the combinations of two different APAP and NP concentrations (APAP: 0.5 and 50 mmol L^−1^, CeO_2_ NP: 10 and 80 µg mL^−1^) of both media were pipetted bubble-free into a cuvette and measured six times each at 37 °C. To investigate background effects, both media were also measured without containing APAP or NP.

### 3.3. Nanoparticle Tracking Analysis (NTA)

NTA measurements were performed with a NanoSight LM20 (NanoSight, Amesbury, Salisbury, UK), equipped with a 632 nm laser. The samples were injected into the sample chamber with sterile syringes. All measurements were performed at room temperature. The samples were diluted in milliQ H_2_O to reach final concentrations of ~10^8^ particles mL^−1^. The software used for recording and analyzing the data was NTA 3.0. All samples (80 µg mL^−1^ CeO_2_ NP and 0.5 and 50 mmol L^−1^ APAP in DMEM) were measured for 60 s at five positions. All measurements were repeated in at least three independent experiments.

### 3.4. Inductively Coupled Plasma Mass Spectrometry (ICP-MS) in Conventional and Single Particle (SP) Mode

For ICP-MS analysis, a quadrupole ICP-MS (iCAP Q, Thermo Fisher Scientific GmbH, Dreieich, Germany) with a PFA ST Nebulizer, a quartz cyclonic spray chamber and a 2.5 mm quartz O-ring-free injector (all from ESI Elemental Service and Instruments GmbH, Mainz, Germany) were used. Gas flows for the plasma, the nebulizer and the auxiliary (all Ar) were set to 14 L min^−1^, 0.89 L min^−1^ and 0.65 L min^−1^ respectively. The flow rate of the sample was 0.4 mL min^−1^. Conventional ICP-MS analysis was performed to determine intracellular CeO_2_ concentrations. The cells were digested using a microwave-assisted acid digestion with 69% HNO_3_ and 30% H_2_O_2_ for 30 min at 200 °C and 160 bar. Measurements were performed in standard mode. For single particle analysis of the NP solutions, the time-resolved analysis (TRA) mode for data acquisition was used. Intensities as a function of time (counts per dwell-time interval) were collected. The acquisition time for each run was set to 60 s with a dwell time (data acquisition rate) of 3 ms. Data were exported to a spreadsheet for further processing following an established procedure according to Pace et al. [29]. Determination of nebulizer efficiency was performed using reference NPs of known sizes as described [29]. We used 60 nm gold reference NPs from the U.S. National Institute of Standards and Technology (NIST, Gaithersburg, MD, USA) RM 8013 as reference NPs. A CeO_2_ NP concentration of 10 µg mL^−1^ was combined with APAP concentrations (0.5 and 50 mmol L^−1^) in DMEM. Based on this, dilutions with a particle concentration of 400 ppt were prepared with ultrapure water.

### 3.5. Cell Lines

The HuH-7 cell line (supplied by ATCC, Manassas, VA, USA) was cultivated in Dulbecco’s modified Eagles medium (DMEM) supplemented with 10% fetal calf serum (FCS, *v*/*v*), 2 mmol L^−1^ L-glutamine, penicillin (100 U mL^−1^), streptomycin (0.1 mg mL^−1^) at 37 °C in humidified atmosphere with 5% CO_2_. After 24 h pre-incubation the cells were exposed to the test substances.

### 3.6. WST-1 Cytotoxicity Assay

In brief, 1 × 10^6^ cells were seeded in 96-well plates for 24 h and then exposed to various concentrations of CeO_2_ and/or APAP. After 48 h of exposure wells were washed using PBS and afterwards WST-1 reagent (Roche, Basel, Switzerland) (0.5 mg mL^−1^) was added and cells were incubated for 2 h at 37 °C and 5% CO_2_. Light absorption of the samples was measured in triplicates on a plate reader (BioTek, Bad Friedrichshall, Germany) according to the manufacturer’s recommendation.

### 3.7. Comet Assay

Slides were incised, coated with 1% (*w*/*v*) basal agarose and numbered consecutively for randomization. In addition, lysis solution, electrophoresis and neutralisation buffers were prepared as described (for details see ESI 1).

In 12 well plates 75 × 10^3^ HuH-7 cells were seeded in a total volume of 1.5 mL per well and incubated before and after exposure to APAP or CeO_2_ NP sample solutions for 24 h at 37 °C and 5% CO_2_. DMEM served as a negative control while methyl methanesulfonate (MMS, Sigma Aldrich, St. Louis, MO, USA) with a final concentration of 43 µmol L^−1^ was the positive control added three hours prior cell harvest.

To minimize damage from the ultraviolet radiation, the following steps were carried out in the dark. Cells were washed with PBS followed by their detachment with 0.5 mL trypsin per well. After 5 min, the reaction was stopped by adding 0.5 mL of ice-cold DMEM to the wells. The contents of the wells were transferred into tubes and stored on ice to minimize DNA repair activities. After cell counting, centrifugation was performed at 4 °C and 300 g for 4 min and the supernatant was removed. Cell count was adjusted to 15 × 10^3^ cells each in 120 mL 0.5% (*w*/*v*) agarose by resuspending the cell pellet in the appropriate amount of DMEM.

After bubble-free application to the ice-cooled slides, the agarose was covered with glass slides, which were removed after solidification of the agarose. Subsequently, the cell lysis was performed by incubating the slides for 72 h with freshly prepared lysis solution at 4 °C. Slides were transferred in random order to the strongly alkaline electrophoresis buffer and incubated for 20 min. Electrophoresis was run for 30 min at 0.89 V cm^−2^ and 450 mA. Afterwards the slides were dipped into neutralization buffer for 10 min at room temperature and stored until further analysis after dehydration in 100% ethanol for 5 min.

Evaluation was performed after fluorescence staining with SYBR gold dye (Sigma Aldrich, St. Louis, MO, USA) using Comet Imager 2.2 (Metasystems, Altlussheim, Germany) software. Slides were excited at 488 nm with 10 × magnification, exposure time of 4.32 s and background correction mode 3. Three slides per concentration with 50 cells per slide were analyzed.

### 3.8. Targeted Metabolomics

The AbsoluteIDQ p180 Kit (Biocrates, Innsbruck, Austria) was used for targeted metabolite profiling as explained by Biocrates and in prior studies [30,31]. The cellular extracts were prepared following the established protocol. For sample analysis a QTRAP 5500 (AB Sciex, Darmstadt, Germany) triple quadrupole mass spectrometer coupled to an Agilent 1200Series high-performance liquid chromatograph (HPLC, Agilent, Waldbronn, Germany) using electrospray ionization tandem mass spectrometry (ESI MS/MS).

Analysis of metabolomics data was done with the help of MetaboAnalyst 5.0 [32].

### 3.9. Imaging Mass Spectrometry—ToF-SIMS

For ToF-SIMS analysis 5 × 10^4^ cells were seeded on 1 cm^2^ silica wafers and cells were allowed to grow for 24 h in the incubator. Afterwards cells were treated with the respective substances for 24 h as described above. The wafers were then washed using 150 mmol L^−^^1^ ammonium bicarbonate solution. Samples were fast frozen and lyophilized prior to ToF-SIMS measurements. For details see elsewhere [18]. Depth profiles were acquired in dual beam mode of a ToF-SIMS V instrument (ION-TOF GmbH, Münster, Germany) of the reflectron-type, equipped with a 30 keV bismuth liquid metal ion gun as primary ion source, a 20 keV argon gas cluster ion source both mounted at 45° with respect to the sample surface and an electron flood gun. Primary and sputter ion currents were directly determined at 200 μs cycle time (i.e., a repetition rate of 5.0 kHz) using a Faraday cup located on a grounded sample holder. A pulse of 0.7 ns from the bunching system resulted in a mass resolution at *m*/*z* < 500 in positive ion mode. The primary ion dose was controlled below 10^12^ ions cm^−2^ to ensure static SIMS conditions. The primary ion gun scanned a field of view of 20 × 20 μm^2^ applying a 512 × 512 pixel measurement raster. Once the primary ion gun was aligned, a ToF-SIMS mass spectrum was generated by summing the detected secondary ion intensities and plotting them against the mass channels. The data were evaluated using the Surface Lab software (ION-TOF GmbH, Münster, Germany).

### 3.10. Statistical Analysis of the ToF-SIMS Data

Statistical analysis of the ToF-SIMS data was performed as described in detail elsewhere [33,34]. In brief, the acquired data were binned to 1 mass unit (u). Data processing was carried out with the statistical package SPSS+ (version 21) (IBM Deutschland GmbH, Ehningen, Germany) using the mass range between 200 mass units and 1200 mass units to detect significant differences.

## 4. Conclusions

The findings presented here highlight the importance of co-exposure assessments for mixtures of pharmaceuticals and NPs. Cytotoxicity investigations demonstrated an altered toxicity profile of APAP upon co-exposure with CeO_2_ NPs. These changes may be the result of complex formation or interactions of APAP with CeO_2_ NPs. The Comet assay results indicated no influence of APAP on the genotoxic potential of CeO_2_ NPs in HuH-7 cells. Therefore, we conclude that the observed toxicity in co-exposure samples is mainly due to APAP metabolites. Cellular uptake analysis of CeO_2_ NPs revealed increased intracellular cerium concentrations upon co-exposure with APAP. Lipidomics and metabolomics data suggest the induction of inflammatory processes via histamine and hydroxyproline signalling. Palmitic acid, a key driver of cellular stress and a pro-inflammatory substance was found to be significantly increased in single and co-exposed APAP samples. These findings in combination with a changed cellular membrane composition and enhanced PI levels indicate that such a co-exposure scenario may result in subsequent fibrosis of the extracellular matrix. The inflammatory processes and membrane alterations may be the reason for the increased uptake in co-exposure samples. Although co-exposure experiments are complex and time consuming, they shed light on substance interactions and the resulting toxicity mechanisms.

## Figures and Tables

**Table 1:** Size, size-distribution and particle number concentration of mixtures of CeO_2_ nanoparticles (NPs) and acetaminophen (APAP) as revealed by dynamic light scattering (DLS) and nanoparticle tracking analysis (NTA), respectively.

Samples	DLS		NTA	
	Z-Average [nm]	PDI	Mode [nm]	Particle Number [Particles mL^−1^]
80 µg mL^−1^ CeO_2_ NP + 0.5 mmol L^−1^ APAP	208	0.291	234.2	1.69 × 10^8^
80 µg mL^−1^ CeO_2_ NP + 50 mmol L^−1^ APAP	211	0.323	256.2	6.68 × 10^7^

## Data Availability

The data presented in this study are available upon justified request.

## Supplementary Materials

The following are available online at https://www.mdpi.com/article/10.3390/ijms22136866/s1.
