# Supplementary material for: Combinatory Effects of Cerium Dioxide Nanoparticles and Acetaminophen on the Liver—A Case Study of Low-Dose Interactions in Human HuH-7 Cells
Source: Int J Mol Sci. 2021 Jun 25;22(13):6866. doi: 10.3390/ijms22136866 (PMC8268126; doi:10.3390/ijms22136866)
Supplement: Supplementary file 1 [file ijms-22-06866-s001.zip › ijms-1250913-supplementary.pdf]

Electronic Supplementary Information

ESI 1: Constituents of different COMET assay buffers.

buffer	substance	Weight or volume
Lysis buffer (2 l)	NaCl<br>Na_2EDTA<br>TRIS<br>H_2O<br>NaOH<br>DMSO<br>TritonX	292.2 g (=2.5 mol l^{-1})<br>74.4 g (=100 mmol l^{-1})<br>2.4 g (=8.25 mmol l^{-1})<br>1800 ml<br>15.5 g (=ca. 200 mmol l^{-1})<br>200 ml (=10% (v/v))<br>20 ml (=1% (v/v))
Electrophoresis buffer (20 l)	Na_2EDTA<br>NaOH<br>Na_2EDTA<br>NaOH<br>deionized H_2O	74.4g (=200 mmol l^{-1})<br>400 g (=10 mol l^{-1})<br>100 ml (=1 mmol l^{-1})<br>600 ml (=300 mmol l^{-1})<br>20 l
Neutralization buffer (2 l)	TRIS<br>HCl37%<br>H_2O<br>HCl/NaOH	97.0 g (=400 mmol l^{-1})<br>58 ml (=ca. 1%)<br>2 l<br>For pH adjustement
TE-buffer	TRIS<br>EDTA<br>deionized H_2O VE<br>HCl/NaOH	1.21 g (=10 mmol l^{-1})<br>0.372 g (=1,3 mmol l^{-1})<br>1 l<br>For pH adjustement

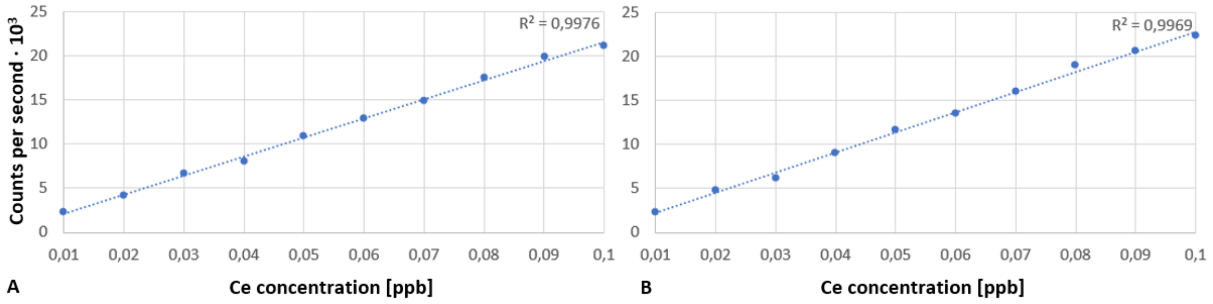

ESI 2: Calibration curves of the concentration of ionic Ce standard in the range 0.01-0.1 ppb. (A) 0.5 mmol APAP in DMEM; (B) 50 mmol/l in DMEM.

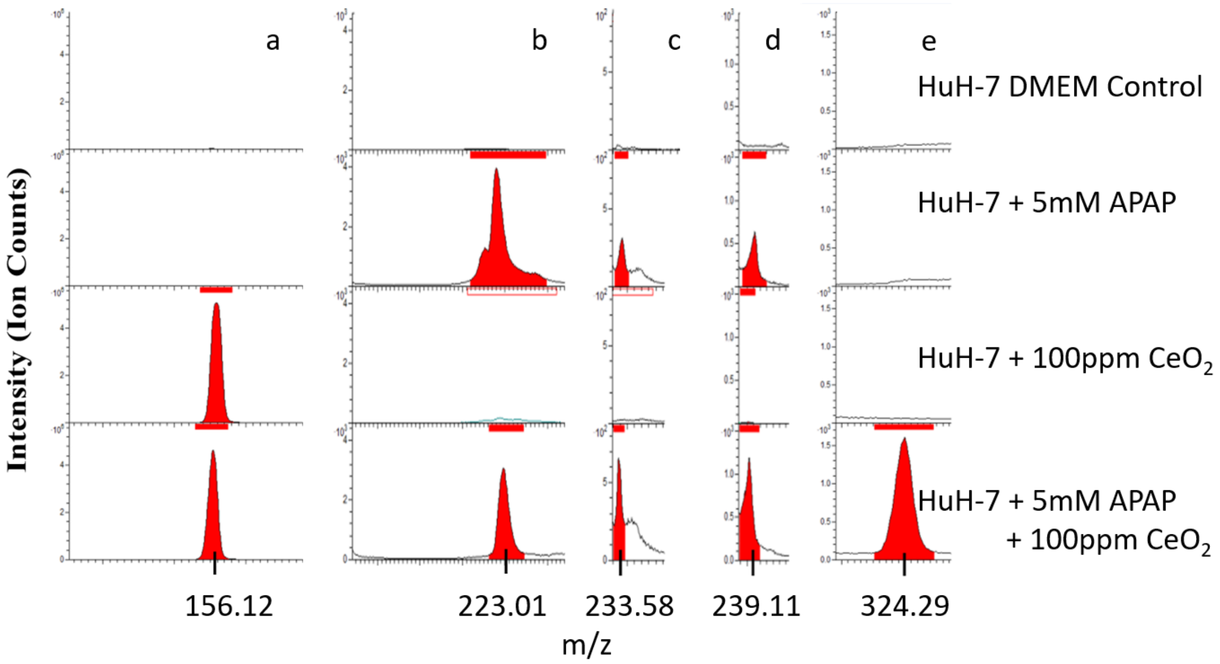

ESI 3: ToF-SIMS mass spectrum (positive ion mode), showing the CeO_2 peak (a, m/z 156.12), the APAP-FeO(OH) peak (b, m/z 223.01), the APAP-Zn$(OH)_2$ peak (c, m/z 233.58) APAP-MnO$(OH)_2$ peak (d, m/z 239.11) and the APAP-CeO(OH) peak (e, m/z 324.29) in red color in HuH-7 liver cells. The first row of spectra show unexposed HuH-7 cells, the second row of spectra show HuH-7 liver cells exposed for 24h with 5 mM APAP, the third row of spectra show HuH-7 liver cells exposed for 24h to 100ppm CeO_2 NPs, the fourth row of spectra show HuH-7 liver cells co-exposed for 24h to 5mM APAP and 100ppm CeO_2 NPs. The x-axis displays the molecular weight; the y-axis corresponds to the ion intensities for the peaks.
